# Rapid Determination of the Geographical Origin of Chinese Red Peppers (*Zanthoxylum Bungeanum* Maxim.) Based on Sensory Characteristics and Chemometric Techniques

**DOI:** 10.3390/molecules23051001

**Published:** 2018-04-24

**Authors:** Xiangqian Yin, Xiaoxue Xu, Qiang Zhang, Jianguo Xu

**Affiliations:** 1 College of Food Sciences, Shanxi Normal University, Linfen 041004, China; m13015425996@163.com (X.Y.); qmping1980@163.com (X.X.); 2 College of Life Sciences, Shanxi Normal University, Linfen 041004, China; zqlf@163.com

**Keywords:** *Zanthoxylum bungeanum* Maxim., geographical origin, sensory characteristics, chemometric techniques, electronic nose, electronic tongue

## Abstract

In this paper, principal component analysis (PCA), linear discriminant analysis (LDAp, artificial neural networks (ANN), and support vector machine (SVM) were applied to discriminate the geographical origin of Chinese red peppers (*Zanthoxylum bungeanum* Maxim.). The models based on color, smell and taste may discriminate quickly and effectively the geographical origin of Chinese red peppers from different regions, but the successful identification rates may vary with different kinds of parameters and chemometric methods. Among them, all models based on taste indexes showed an excellent ability to discriminate the geographical origin of Chinese red peppers with correct classifications of 100% for the training set and the 100% for test set. The present study provided a simple, efficient, inexpensive, practical and fast method to discriminate the geographical origin of Chinese red peppers from different regions, which was of great importance for both consumers and producers.

## 1. Introduction

Chinese red pepper (*Zanthoxylum bungeanum* Maxim.) is an aromatic tree and shrub plant belonging to the genus *Zanthoxylum* of the family Rutaceae and native to eastern China that is now mainly distributed in Hebei, Shanxi, Shaanxi, Sichuan, Gansu, and Shandong provinces of China and some Southeast Asian countries. It grows quickly and matures after three years, yielding fruit that consists of *Z. bungeanum*, namely, the red shell, which is an important spice, and dark seed, a side product of *Z. bungeanum*. The fruits of this species are the most popular huajiao commercial product, called ‘da hong pao’ (big red robe). Chinese red pepper is often used as a kind of traditional Chinese medicine for treatment of vomiting, toothache, stomach ache, abdominal pain, eczema, and diarrhea [1,2]. There are several papers published about the components and different bioactivities including antioxidant activity, antitumor activity, anti-inflammatory activity, antimicrobial activity and insecticidal activity of the pericarps of *Z. bungeanum* Maxim [3,4,5,6]. The pericarp of the fruits of *Z. bungeanum* has been widely used as a well-known pungent food condiment because they have a distinctive aroma that can be generally described as fresh, floral, spicy, and green. Because of difference in preferences, consumers focus more on the geographical origin of red peppers because red peppers from different areas have different characteristics including color (red or green), aroma and taste “ma la xiang” (tingling, chili and savory) [7,8], which can also affect the standardization and quality stability of food. Therefore, it is very important for consumers and producers to distinguish quickly and accurately the geographical origin of red pepper. But as far as we know, there have been few reports of its geographical origin traceability.

Supervised pattern recognition techniques have been applied to a wide variety of chemical data (chromatographic, spectrometric, spectrophotometric, spectroscopic, sensorial, et al.) with diverse purposes such as profiling, fingerprinting, authentication, detection of adulteration, food quality assessment, determining the geographical origin, data interpretation, et al. [9]. However, chemical component analysis was time-consuming, complicated, tedious, and the process needed plenty of manpower, material and financial resources in order to gain these chemical data. In this paper, *Z. bungeanum* cultivated from five major production areas including Hanyuan (HC), Ruicheng (RC), Wudu (WD), Hancheng (HC), and Maoxian (MX) regions in China were collected and their sensory characteristics such as color, taste and smell were analyzed and compared, and further determined the geographical origin based on different supervised pattern recognition techniques including principal component analysis (PCA), linear discriminant analysis (LDA), back propagation artificial neural networks (BP-ANN) and support vector machine (SVM) analysis. The aim of this study was to provide a rapid and efficient method for distinguishing the geographical origins of Chinese red pepper from different regions.

## 2. Results

### 2.1. Discriminant Analysis Based on Color

Profiles of colors of Chinese red pepper pericarp and powder were shown in Table 1. The red pepper pericarp from Ruicheng (RC) had the highest red (R) and green (G) values, while there is no difference in R and G values among other samples. The blue (B) value from Maoxian (MX) pericarp was higher than others, but no significant difference was found among samples from different origins. Similar to pericarp color, the red pepper powder from RC had the highest lightness (L *) and greenness (b *) values, and the lowest redness (a *) value; Hanyuan (HC) pepper powder exhibited the lowest L * and b * values. There were some differences in colors for MX, HC, Wudu (WD), Hancheng (HC) origins, but they can't be completely separated each other.

In the present study, a training set with known class memberships is used to calculate a classifier. A test set, containing objects not included in the training and also with known class memberships, served to validate the model built. The selected multivariate methods including ANN, LDA, and AVM were used to construct models for rapid determining the geographical origin of Chinese red pepper, and the discrimination results were shown in Table 2. Four models exhibited different degrees of success, ranging from 78.8 to 93.9% for the training set (AC-tr) and from 86.7 to 93.3% for the test set (AC-te) based on pericarp color. Among them, the ANN was the best, with an average successful identification rate of 93.9% for the training set and 86.7% for the test set. The discriminant models based on powder color showed a degree of accuracy range from 69.7 to 100% for the training set and from 86.4 to 93.3% for test set, which demonstrated the some discriminatory ability to differentiate the five origins. The ANN exhibited a much higher distinguish effect with an identification rate of 100% for the training set and 93.3% for the test set.

### 2.2. Discriminant Analysis Based on Smells

#### 2.2.1. Response Curves of Electronic Nose

**Figure 1:**
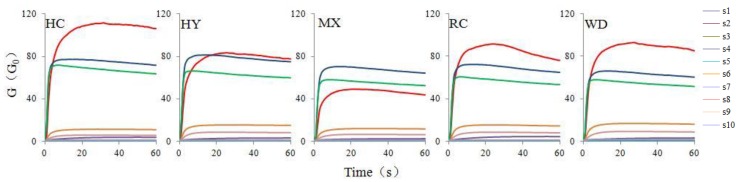
Typical responses of Chinese red peppers obtained by direct e-nose measurement.

Figure 1 shows the typical responses of direct electronic nose measurement during the measurement of different samples. Each curve represented the change of a sensor’s ratio of conductance during measurement. The x-axis represents time, and the y-axis represents the sensor’s ratio of conductance of the electronic nose. Each curve represented the change of a sensor’s ratio of conductance during measurement. As shown in Figure 1, the conductivity of the all sensors began to increase rapidly reached the maximum, and finally reached stable equilibrium after 20th second. Different sensors had different response values for various samples, and there had remarkable differences in the conductivity of some sensors such as S2, S7, S9 sensors for samples from different origins. In this paper, the response values of the 30th second of each sensor were extracted and analyzed in the following study.

#### 2.2.2. PCA Analysis

**Figure 2:**
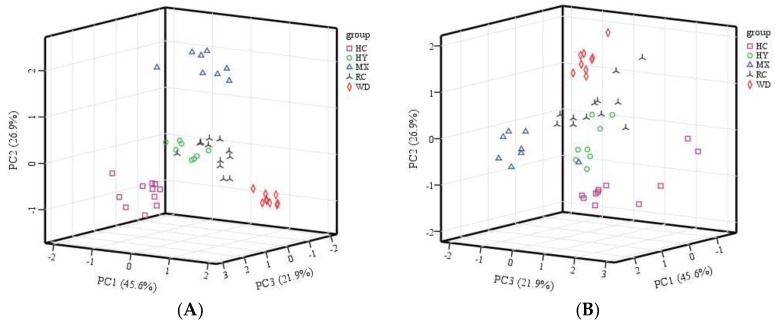
Three-dimensional principal component score plot using the first three score vectors (**A**) front view and (**B**) rear view.

At the preliminary stage, PCA was firstly carried out on the basis of the conductivity proportion of each sensor in ten sensors for exploratory analysis in order to verify the significance of the different geographical origins of the samples. Figure 2 showed the three-dimensional score plot in which separation amongst geographical origins of Chinese red pepper was not complete. Three principal components were extracted from 10 variables by PCA, and explained 94.4% of the total variance. The first principal component represented 45.6%, and the following principal components represented 26.9% and 21.9% respectively, which made differentiation clearer. From Figure 2, we can see that most samples can still be correctly distinguished, inferring that they had inherent compositional differences and determining the geographical origin was feasible. However, in this plot there was a certain area of overlapping in which no clear differentiation could be made between Hanyuan (HY) and Ruicheng (RC) cities. Therefore, different methods of LDA, ANN and SVM were utilized in the following studies for actual discrimination. 

#### 2.2.3. Evaluation of Geographic Origin

Table 3 summarized the results obtained after the application of the different classification models. The discriminant models based on smells of red pepper pericarp had a degree of accuracy range from 87.9 to 97% for the training set and from 93.3 to 100% for test set, indicating a satisfactory performance of this model for the classification of red pepper samples from different origins. Of the four techniques, the performance of the SVM linear model exhibited better distinguish effect with a success rate of 90.9% for the training set and 100% for the test set, follow by LDA and ANN, the lowest for SVM-RBF.

### 2.3. Discriminant Analysis Based on Tastes 

#### 2.3.1. Profiles of Tastes 

**Figure 3:**
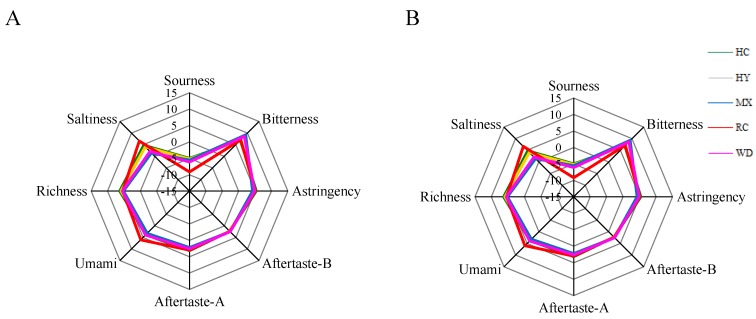
Radar maps for the sensory score of samples based on the electronic tongue (**A**) 40 °C and (**B**) 80 °C.

Figure 3 showed the tastes of red pepper samples from different origins when the steeping temperature was at 40 °C and 80 °C. It was found that the taste index of red pepper was abundant, and all the sensors had a great response to soaking solution of the red pepper by the electronic tongue. The sensors were sourness, bitterness, astringency, aftertaste-B, aftertaste-A, umami, richness and saltiness, and there were some differences in some taste indexes for different samples. Different steeping temperature can have certain influence on tastes. No obvious change was found in umami, aftertaste-A, aftertaste-B and astringency between 40 °C and 80 °C. However, and the higher temperature improved sourness, saltiness, richness, but declined the bitterness.

#### 2.3.2. Evaluation of Geographic Origin

Different methods of LDA, ANN and SVM were used to evaluate the geographic origin of red pepper samples, and the results at 40 °C were summarized in Table 4. It is clear that every red pepper geographical origin was correctly classified by the all models, and the accuracy rates of four discriminant models based on the tastes of red pepper pericarp reached to 100% for both the training set and test set in the classification of the five red peppers’ geographical origin. The same discriminant results were reached at 80 °C (no shown), indicating that temperature did not affect the discriminant, although temperature had a certain influence on the taste. These results showed that all of LDA, ANN and SVM methods can quickly and completely distinguish the red pepper from different origins based on their tastes, which also indicated that the electronic tongue was a useful tool for to differentiate the geographical origin of Chinese red peppers.

**Figure 4:**
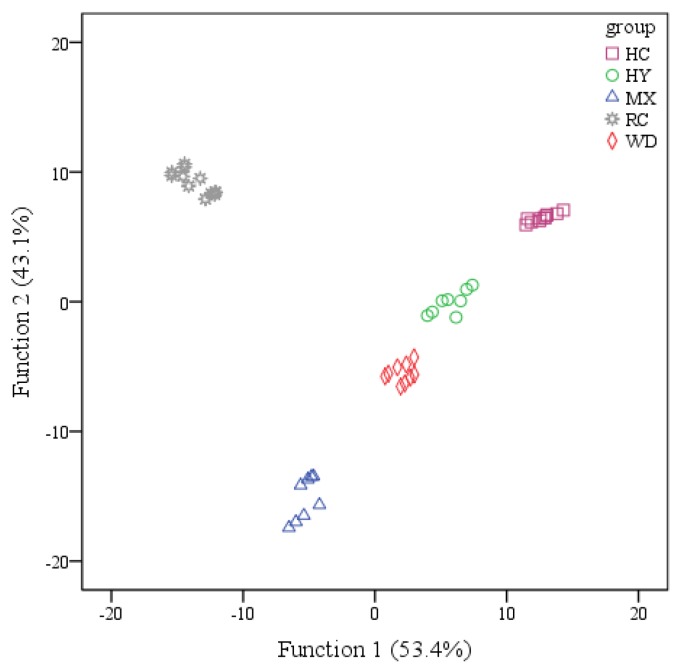
Scatter plot of Chinese red peppers from different regions based on the two discriminant functions.

LDA is based on the determination of linear discriminant functions, which maximize the ratio of between-class variance and minimize the ratio of within-class variance, and LDA is probably the most frequently used and studied supervised pattern-recognition method [9,10]. For this reason, using the stepwise method, LDA was carried out to establish functions, and the statistical significance of each discriminant function was evaluated on the basis of the Wilks’ lambda criterion. From Figure 4, six variables (bitterness, astringency, aftertaste-b, aftertaste-a, umami, saltiness) were selected and thought to contribute significantly to the ability for discriminating the geographical origin, and four discriminant functions were constructed. The first two functions explained 96.5% of the variance (Function 1 explained 53.4% of the total variance, and Function 1 explained 43.1%). It is clearly shown that Chinese red pepper from different regions was well distinguished from each other, confirming that selected variables provided the useful information for Chinese red pepper classification. Discriminant functions are shown as follows,

F1 = −1.322 − 4.060 Bitterness + 1.224 Astringency + 19.328 Aftertaste-B + 5.121 Aftertaste-A − 4.070 Umami + 1.209 Saltiness

F2 = 0.822 − 4.086 Bitterness+ 5.269 Astringency + 9.540 Aftertaste-B − 5.844 Aftertaste-A + 0.618 Umami + 1.684 Saltiness

F3 = 2.167 + 9.852 Bitterness − 10.712 Astringency − 34.227 Aftertaste-B + 23.422 Aftertaste-A − 2.961Umami + 3.548 Saltiness

F4 = 1.718 + 11.481 Bitterness − 1.550 Astringency − 50.346 Aftertaste-B + 17.530 Aftertaste-A − 6.024 Umami + 0.389 Saltiness

## 3. Discussion

Nutritive value as well as the bioactivity components from plant-derived products were greatly influenced by geographic origins [11,12]. For these reasons, determination of geographical origin authenticity of food and agricultural products was very important for both consumers and producers, and there have been numerous efforts and methods to certify the geographical origin of food and plant products [11,12,13]. Some studies determined the geographic origin of some agricultural products based on the combination of chemometrics and the elements [11,14,15,16], amino acids [17,18], phenolic compounds [19,20,21], and physicochemical parameters [20,21,22,23] with different degrees of success. However, component analysis was time consuming, complicated, tedious, and required chemical use which was sometimes harmful to the environment. The process needed not only plenty of manpower, material and financial resources, but also slow determining speed and low efficiency. Besides, sensory indexes such as color, shape, smell, and taste were considered to be effective and fast indicators of determining the geographical origin, because in fact the sensory characteristics of agricultural products were exactly the integrative and external reflections of chemical components associated with it. For example, the electronic nose or electronic tongue does not measure the quality indexes directly; it actually measures volatiles or other soluble compounds that are well-correlated with the quality indexes [24]. More importantly, these sensory parameters were easier to measure compared with component analysis. Some researchers applied data mining techniques to successfully determine the geographical origin of some food and agricultural products based on their sensory characteristics such as color, shape, size, taste, and smell by image analysis, electronic nose and electronic tongue system respectively [25,26,27,28,29,30], which confirmed further the feasibility of this method.

The characteristics of plant-derived products can be highly influenced by several environmental and geological factors such as soil type, soil parent material, water, soil pH, and climate conditions. The contents and constituents of Chinese red peppers from different species or areas differed significantly because of environmental growth influences [5,7,8,31,32,33], leading to the differences in the sensory characteristics of Chinese red peppers from different areas, which was very important for consumers and producers. Hancheng (HC), Hanyuan (HC), Maoxian (MX), Ruicheng (RC), and Wudu (WD) regions belong to warm temperate continental monsoon, subtropical humid monsoon, subtropical monsoon, warm subhumid continental, and north subtropical sem-humid climate, respectively. In addition, the main type of soil planting tobacco in Hanyuan and Wudu was yellow-brown soil, while the main soil types are brown, dark brown, and cinnamon soil in Hancheng, Maoxian, and Ruicheng regions respectively. These differences provided the feasibility for distinguishing the geographic origin of Chinese red peppers. In this study, we first represented the approach to distinguish the geographical origin of Chinese red peppers based on the sensory characteristics and using the LDA, ANN and SVM methods, and obtained better discriminant results as a whole. Nevertheless, the identification rate was concerned with different kinds of sensory parameters and chemometric methods. Compared with other methods, discrimination methods built in this study were not just simple, rapid, high efficient, low expenditure, no pollution and but also more practical and suiting for spot measuring.

## 4. Experimental Section

### 4.1. Plant Materials

The 48 samples of dried *Z. bungeanum* pericarp which were planted in 2016 were collected in November 2016 from Hanyuan (HC), Ruicheng (RC), Wudu (WD), Hancheng (HC), and Maoxian (MX) regions, China. Previously, these samples were distributed uniformly as a thin layer on the trays and dried under direct sunlight at temperatures between 16 °C and 28 °C in local area after harvests, and the moisture content was about 7.2–8.1% for samples. The other detailed information was listed in the Table 5. These raw pericarps were labeled according to their sources, and were stored in bottles at −18 °C until analyses.

### 4.2. Pericarp Color Analysis

According to the method previously described with some modifications [34], placed 40 Chinese red peppers down on the glass tray of high speed color document scanner DS-60000 (Epson (China) Co., Ltd., Beijing, China), respectively. Covered it, and started the software to scan and get photos of red pepper from five areas. Imported the photos into the Photoshop CS6 (Adobe Systems Incorporated, San Jose, CA, USA) to get the data of red (R), green (G), blue (B) as the analysis data.

### 4.3. Powder Color Analysis

The dried pericarp of Chinese red peppers were ground into powders with a mixer and filtered with 50 mesh numbers. The color was measured through the CIE L * a * b * system using a Minolta Chroma Meter CR-330 (Minolta, Ramsey, NJ, USA). During measurement, CIE L *, a * and b * values were obtained, representing lightness (L *), redness (a *) and greenness (b *). The results reported are the average of at least 10 replications.

### 4.4. Smell Analysis

The PEN3 electronic nose (Airsense Analytics, Schwerin, Germany) was used to discriminate odor patterns of different aroma models. The sensor array of this analytical instrument was composed of ten different metal oxide sensors (MOS) positioned in a small chamber. The condition was as following: each sample (80 g of pericarp) was placed in a 500-mL airtight glass vial beaker that was sealed with plastic wrap, and equilibrated for 15 min (headspace-generation time), respectively. During the measurement process, the headspace gaseous compounds were pumped into the sensor arrays through Teflon tubing connected to a needle in the plastic wrap, causing the ratio of conductance of each sensor to change. The measurement phase lasted for 60 s, which was long enough for the sensors to reach stable signal values. The signal data from the sensors were collected by the computer once per second during the measurements. After each measurement, zero gas (air filtered by active carbon) was pumped into the sample gas path from the other port of the instrument for 60 s (flush time). All the electronic nose measurement procedures were carried out at a temperature of 25 ± 1 °C. Each analysis was repeated ten times.

### 4.5. Taste Analysis

The five basic tastes (acid, sweet, bitter, salty, and fresh) and evaluation of astringency was conducted with the taste sensing system TS-5000Z (Intelligent Sensor Technology, Inc., Kanagawa, Japan) consisting of reference electrodes, multichannel lipid/polymer membrane electrodes, an auto-sampler, an electronic unit for data acquisition, and a personal computer with an advanced chemometric software package. Each sample was measured after the electric potentials of all membranes had been stabilised in standard solutions. Firstly, 10 g of each pericarp sample was steeped with 100 mL of distilled water for 30 min at 40 °C and 80 °C, respectively. After *filtration,* the supernatants were collected, cooled to room temperature, and then dilute with distilled water to 100 mL. Then, the sensor array was immersed into the sample solution, and the response signals at the equilibrium state were collected as variables for statistical analysis. Each sample was measured, and ultra-pure water was used to clean the sensors before each subsequent measurement, to ensure that stable potentials were obtained before detecting the next sample. Each sample was measured in five times, the first two measurement cycles were discarded due to instability, and the rest three stable sensor responses were obtained and averaged. The mean value of the three replicated measurements was used for subsequent analysis. 

### 4.6. Multivariate Data Processing

One-way analysis of variance was first carried on each single component of all the samples to determine significant differences (*p* < 0.05). Evaluation of the geographical origin of fruits can be facilitated using multivariate approach. Principal component analysis (PCA) is used to previsualize data trends. Linear discriminant analysis (LDA), artificial neural networks (ANN), support vector machine (SVM) were applied to carry out the classification of samples according to their geographical origin. SVM need to optimize several parameters in such a way that a suitable number of parameters are selected to build the model. In this work, penalty factor C, ε of the ε-insensitive loss function and kernel type for SVM, were calculated by using a tenfold cross-validation technique by which maximum accuracy is selected. In addition, we ran the SVM with two types of kernel function: linear and RBF.

## 5. Conclusions

In conclusion, the characteristic parameters of color, smell and taste were determined from samples of Chinese red peppers by using image analysis, an electronic nose, and an electronic tongue system. The chemometric methods—including LDA, ANN and SVM—found that combining sensory characteristics may discriminate the geographical origin of Chinese red peppers quickly and effectively from different regions, but the successful identification rates may vary with different kinds of parameters and chemometric methods. Among them, four models exhibited different degrees of success, ranging from 78.8 to 93.9% for the training set, and from 86.7 to 93.3% for the test set based on pericarp color; the discriminant models based on powder color showed a degree of accuracy range from 69.7 to 100% for the training set and from 86.4 to 93.3% for test set; the discriminant models based on smells of red pepper pericarp had a degree of accuracy range from 87.9 to 97% for the training set and from 93.3 to 100% for test set, while all models based on taste indexes showed an excellent ability to discriminate Chinese red peppers with correct classifications of 100% for training set and 100% for test set. The present study provided a simple, efficient and fast method to discriminate the geographical origin of Chinese red peppers from different regions, which was of great importance for both consumers and producers.

## Figures and Tables

**Table 1:** Color parameters of red pepper pericarp and powder from different origins.

Samples	Pericarp			Powder		
	R	G	B	L *	a *	b *
HC	93.9 ± 2.7 ^ab^	63.7 ± 3.1 ^b^	58.8 ± 1.9 ^a^	30.4 ± 4.4 ^b^	16.5 ± 0.6 ^a^	22.4 ± 1.3 ^b^
HY	81.9 ± 4.6 ^b^	59.9 ± 2.3 ^b^	58.4 ± 1.8 ^a^	37.2 ± 4.3 ^ab^	15.3 ± 0.6 ^ab^	26.1 ± 1.4 ^b^
MX	85.9 ± 3.9 ^b^	63.7 ± 4.7 ^b^	63.9 ± 4.0 ^a^	37.9 ± 4.5 ^ab^	16.9 ± 0.5 ^a^	22.8 ± 1.3 ^b^
RC	104.6 ± 4.3 ^a^	77.7 ± 4.7 ^a^	62.6 ± 2.9 ^a^	46.2 ± 5.6 ^a^	13.7 ± 0.5 ^b^	34.7 ± 3.3 ^a^
WD	88.9 ± 6.5 ^b^	63.7 ± 3.3 ^b^	62.3 ± 2.2 ^a^	44.1 ± 6.5 ^ab^	14.5 ± 0.9 ^b^	27.0 ± 3.4 ^b^

Numbers represent mean values of ten independent replicates ± SD. Different letters within a column indicate statistically significant differences between the means (*p* < 0.05).

**Table 2:** Discrimination results (accuracy rate) of different models by color.

Groups	Number of Samples		LDA		ANN		SVM			
							RBF		Linear	
	Training Set	Test Set	AC-tr (%)	AC-te (%)	AC-tr (%)	AC-te (%)	AC-tr (%)	AC-te (%)	AC-tr (%)	AC-te (%)
Pericarp color										
HC	7	4	85.7	100	100	100	71.4	100	85.7	100
HY	6	2	100	100	100	100	100	100	100	100
MX	6	2	66.7	50	83.3	100	66.7	100	66.7	100
RC	8	4	100	100	100	100	100	100	100	100
WD	6	3	66.7	66.7	83.3	33.3	50	66.7	33.3	66.7
	total		84.8	86.7	93.9	86.7	78.8	93.3	78.8	93.3
Powder color										
HC	7	4	42.9	100	100	100	57.1	100	57.1	100
HY	6	2	57.1	100	100	50	100	100	83.3	100
MX	6	2	100	100	100	100	66.7	100	50	100
RC	8	4	100	75	100	100	100	100	100	100
WD	6	3	83.3	66.7	100	100	83.3	66.7	83.3	66.7
	total		69.7	86.7	100	93.3	78.8	93.3	69.7	93.3

**Table 3:** Discrimination results (accuracy rate) of different models by electronic nose.

	Samples	Number	LDA (%)	ANN (%)	SVM	
					RBF (%)	Linear (%)
Training set	HC	7	100	100	100	100
	HY	6	83.3	83.3	66.7	50
	MX	6	100	100	100	100
	RC	8	100	100	100	100
	WD	6	100	100	100	100
	total		97	97	87.9	90.9
Test set	HC	4	100	100	100	100
	HY	2	100	100	50	100
	MX	2	100	100	100	100
	RC	4	75	75	100	100
	WD	3	100	100	100	100
	total		93.3	93.3	93.3	100

**Table 4:** Discrimination results (accuracy rate) of different models by electronic tongue.

Groups	Number of Samples		LDA (%)		ANN (%)		SVM RBF (%)		SVM Linear (%)	
	Training Set	Test Set	Training Set	Test Set	Training Set	Test Set	Training Set	Test Set	Training Set	Test Set
HC	7	4	100	100	100	100	100	100	100	100
HY	6	2	100	100	100	100	100	100	100	100
MX	6	2	100	100	100	100	100	100	100	100
RC	8	4	100	100	100	100	100	100	100	100
WD	6	3	100	100	100	100	100	100	100	100

**Table 5:** Geographical sources of Chinese red peppers.

Sample	Number of Samples	Longitude (E)	Latitude (N)	Climate Type	Agrotype
HC	11	E110°7′–110°37′	N35°18′–35°52′	Warm temperate continental monsoon	brown
HY	8	E102°16′–103°00′	N29°05′–29°43′	Subtropical humid monsoon	yellow brown
MX	8	E102°56′–104°10′	N31°25′–32°16′	Subtropical monsoon	dark brown
RC	12	E110°36′–110°42′	N34°36′–34°48′	Warm sub-humid continental	cinnamon
WD	9	E104°34′–105°38′	N32°47′–33°42′	north subtropical semi-humid	yellow brown
